# Non-Invasive Prenatal Testing in Germany

**DOI:** 10.3390/diagnostics12112816

**Published:** 2022-11-16

**Authors:** Thomas Liehr, Tigran Harutyunyan, Heather Williams, Anja Weise

**Affiliations:** 1 Jena University Hospital, Friedrich Schiller University, Institute of Human Genetics, 07747 Jena, Germany; 2 Department of Genetics and Cytology, Yerevan State University, Yerevan 0001, Armenia; 3 Tempus Labs, Inc., Chicago, IL 60654, USA

**Keywords:** first trimester-screening (FTS), teratogen effects, multigenetic diseases, pregnant woman perspective, false-positive, false-negative, knowledge of specialists and public

## Abstract

In the short 10 years following the introduction of non-invasive prenatal testing (NIPT), it has been adapted in many countries around the world as a standard screening test. In this review, this development was analyzed with a special focus on Germany. As a result, it can be stated that all known advantages of NIPT apart from “compensating for having no access to centers offering invasive diagnostics” are valid for Germany. In addition, following a review of the international literature, all documented issues with NIPT are also observed in Germany. However, the German Gene Diagnostics Act (GenDG) addresses a number of these issues, for example, the regulations by GenDG hamper induced abortions, based exclusively on an abnormal NIPT result. At the same time, GenDG has created new problems, as a possible collusion between the “right not to know with regard to parts of the examination result” may occur, or that the sex of the fetus must not be reported to the pregnant woman before the 12th week of gestation. Main conclusions drawn are that appropriate training and the continuing education of the physicians providing NIPT-related counseling are needed, as well as the provision of balanced and comprehensive information for the pregnant woman or the couple that is imperative.

## 1. Introduction

During the last five decades, a dream of humankind has come true; what was previously purely science fiction is now reality: owing to the technical progress over this period it became routine and commonplace that pregnant women could obtain sound “prenatal information”, regarding the health of their unborn child [1]. At first, there is the opportunity to visualize the unborn child by ultrasound imaging [2]. While progress in sonographic approaches were already striking [2], the fast developments of (cyto-) genetics and genomics opened new and previously unexpected ways to even obtain information, concerning the genetic status of a fetus [1]. There are two major principle modalities to perform prenatal genetic diagnostics: either by (i) invasive or by (ii) non-invasive means [3].

Invasive prenatal genetic diagnostics depends on fetal or placental tissues, which can only be acquired by approaching the unborn with a needle to gain this material for further in vitro studies. Conversely, non-invasive tests do not disturb the fetal or placental tissues at all; they depend on sonography, on proteins or free placental DNA accessible from the maternal blood. While all, so-called “non-invasive prenatal diagnostic approaches,” are in reality screening tests, it is only invasive tests, based on fetal tissue that can provide clear answers on whether the unborn has a specific genetic condition or not [1,2,3]. 

All invasive prenatal tests involve a certain risk for the fetus. In the 1980s to 1990s, chorionic villi sampling (CVS) from placenta, amniocentesis (AC) or umbilical cord blood (UCB) acquisition had an abortion risk between >1% and 3% [4], which dropped to 0.5% to 1% by the 2010s [5]. Since then, the application of needles with smaller diameters has reduced these rates down to between 0.1% and 0.3% [3,4,6]. Nonetheless, using German Google search (Supplementary Figure S1), the old figures from the 1980s show up at first [7]; and even still, peer reviewed papers, as recent as 2022 refer to these long outdated statistics [8]. 

The aforementioned initially relatively high-risk figures of invasive approaches [4,5] stimulated substantial medical research, which developed many tools for non-invasive prenatal diagnostics (for the currently available invasive and non-invasive prenatal screening tests by week of gestation (w.o.g.) see Table 1 [1,4,5,8,9]).

The newest non-invasive prenatal diagnostic test approach is the so-called non-invasive prenatal testing (NIPT), also referred to as non-invasive prenatal screening (NIPS) or non-invasive prenatal diagnostics (NIPD). However, the wording NIPD should not be used, as NIPT is clearly a screening test [1,2,3]. As highlighted in Table 1, the only non-invasive approach assessing fetal tissues is the study of fetal nucleated erythrocytes from the maternal blood [9]. The latter is a promising idea, still hampered at present, mainly by the fact that reliable and efficient isolation tools for fetal nucleated erythrocytes are not yet available [10]. However, single reports prove that molecular [11] and even molecular cytogenetic analyses can be completed, based on these fetal blood cells [12]. 

## 2. Technical Bases of NIPT

NIPT is based on free placental DNA, intentionally and misleadingly referred to as ‘cell free fetal DNA = cffDNA’ in NIPT-related literature, from the very beginning [13]. This cffDNA can be reliably detected in the blood of a pregnant woman at approximately 10 w.o.g.; the cffDNA concentration rises during pregnancy and disappears completely from the maternal blood serum only hours after birth [8]. The latter is in contrast to fetal lymphocytes, which can circulate for decades in the maternal body, while nucleated erythrocytes normally die within the range of weeks [9].

Technically, NIPT is predominantly based on whole genomic sequencing (WGS), either shotgun massively parallel sequencing (s-MPS), targeting massively parallel sequencing (t-MPS) or single nucleotide polymorphism (SNP) based WGS. In MPS based approaches, the maternal and placental DNA cannot be distinguished; aneuploidies are recognized as changes in copy numbers for the studied chromosomal regions. Only the SNP-based approaches allow the separation of cffDNA from the maternal, serum derived DNA, and also allow for the detection of triploidy [14]. In addition, the combination of WGS with the real-time polymerase chain reaction [15] or target enrichment [16], and NIPT based on microarray-technology or rolling circle amplification [17] are reported in the literature, but are not widely used as of yet.

A major problem of the publications reporting on NIPT, is that these technical differences between platforms are not discussed extensively [14,18]. NIPT results are reported for in-house developed or the application of commercially available platforms, and the comparison of the results is achieved interchangeably, as if differences in platforms and their technical restrictions do not matter.

Apart from these problems, NIPT-studies can include testing for

(a) trisomy 13, 18 and 21, only;
(b) aforementioned trisomies and for changes in the copy numbers of sex chromosomes;
(c) all mentioned in b) plus only DiGeorge syndrome [19];
(d) all mentioned in b) plus other selected microdeletion or microduplication syndromes [20];
(e) all mentioned in (b), (c) or (d) plus all other copy number changes of all other autosomes;
(f) whole genome, for any kind of copy number alteration.
(g) Finally, some NIPT providers have started to also offer screening for point mutations of specific genes, such as the Rhesus factor and blood groups.

Overall, there are dozens of variants of NIPT available, but they are not really distinguished in the literature [18,21]. This combination issue, if applied to all studies using molecular cytogenetics/fluorescence in situ hybridization (FISH) in prenatal samples [22], would be as if they were all reported as similar results e.g., “invasive prenatal FISH testing” (IPFT), a misnomer, which would fail to distinguish whether the results were based on interphase or metaphase FISH, and/or which and how many different probes were applied. This exaggerated comparison is merely intended to illustrate what is being lumped together in the literature within the NIPT field.

## 3. NIPT and Its Advantages

The idea to perform NIPT, based on WGS is indisputably brilliant. The concept to study cells from the placenta without bothering or endangering the developing individual is laudable. Screening the literature for bolstering-points for NIPT include conclusions that the platform:

I. can be applied earlier than other tests during pregnancy;
II. has the potential to reach populations with no access to centers offering invasive diagnostics [23];
III. can exclude and detect a trisomy 21 with the highest probability of all non-invasive approaches—the positive predictive value (PPV) in cases of a NIPT suggesting trisomy 21 is >99%;
IV. can bring a psychological relief to an anxious pregnant woman in the case of a normal NIPT result; and
V. can lead to an early pregnancy termination in cases of an early detection of an adverse genetic condition via NIPT [8,14,18].

Interestingly, these are the only five points to identify in the literature as advantages of NIPT. Moreover, as outlined in the next chapter, the interpretations of NIPT results are either not really understood in detail and/or at the least not well communicated [14,18]. Further compounding the issue, the advantages, and restrictions of NIPT may be unknown to both the MDs ordering the test, and the pregnant women taking the test [24].

## 4. NIPT and Its Practical Restrictions and Shortcuts

Some of the problems of NIPT have already been reviewed in this paper and were recently summarized by our group [14,18]; thus, herein, only the main keywords are repeated and summarized in one to two sentences. The supporting evidence can be found in references [14,18].

**Figure 1:**
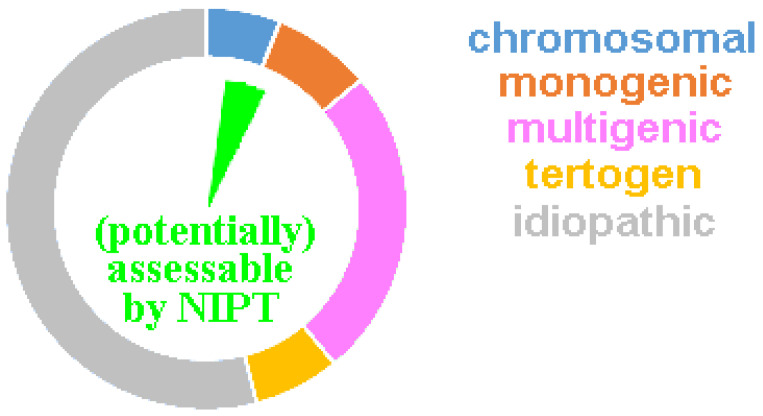
The ring diagram includes the 3–6% of newborns with major inborn abnormalities. A chromosomal disorder is present in ~6%, teratogenic damage in ~7%, and a monogenetic or multigenetic disease in ~8% or ~25%, respectively. For the remaining ~54%, the diagnosis usually remains a lifelong suffering attributed to an “idiopathic disorder”, i.e., the cause remains unclear. The green inner pie diagram shows the ~5 to max 10% of cases potentially assessable by NIPT.

(a) The term NIPT suggests to the uninformed public that the platform studies cell free fetal DNA (cffDNA); however, all cell free DNA in the pregnant individual’s blood (not being of maternal origin) derives from the placenta (and not the fetus); thus, in 1–2% of the cases studied by NIPT, a placenta confined mosaic condition must be expected. They constitute a part of the false positive and negative NIPT results [14,18].
(b) Each abnormal NIPT result has to be checked by an invasive prenatal test, optimally an AC, as indicated by each commercial NIPT provider in the package leaflet [8]. However, one major motivation to do NIPT is to avoid any invasive procedure, due to the previously suggested high abortion risk [3,4,5,6].
(c) A normal NIPT result can maximally exclude (to a certain extent) genetic conditions, which are covered by the NIPT platform used. A normal NIPT is never synonymous with the statement: ‘a healthy child will be born’ [25]. As recently shown, NIPT can at best detect 5–10% of all cases potentially born with birth defects [18] (Figure 1).
(d) 90% of aneuploid fetuses detected in NIPT would be aborted naturally and do not survive to birth [18]. Thus, 90% of pregnant women with a true abnormal NIPT result would not have to undergo an induced abortion.
(e) 5–10% of NIPTs provide no result at the first attempt—this is most often due to the low fraction of cell-free placenta derived DNA. The greater the obesity severity in pregnant women, the more likely a ‘no-call’ result will occur [14,18]. The number of cases repeated is not extensively reported in the literature, which could yield data on how many of these cases ultimately yield an informative result. However, it should be noted that within this population, there is overrepresentation of cases ending in an early abortion, largely attributed to a placenta that is too small for the gestational age and preeclampsia.
(f) False positive NIPT results can be due to confined placental mosaicism, a vanishing twin, a maternal sex chromosome mosaic, or a maternal undetected tumor [8,14,18].
(g) There can be unexpected findings, even for NIPTs evaluating only trisomies 13, 18 and 21. Partial trisomies or even tetrasomies, e.g., supernumerary isochromsome 18p syndrome can be picked up: several publications report this surprise detection in the literature (e.g., [26,27,28]).
(h) As aforementioned, the PPV for trisomy 21 is >99% in all available NIPTs; however, it is neither known nor studied why screening for trisomies 13 and 18, is less reliable. PPVs for all other tested copy number changes (including sex chromosomes, other autosomes, and microdeletion/-duplications) remain substantially lower between 5 and 60—which means 40 to 95% of women receiving an abnormal NIPT result have indeed received a false positive result [18].
(i) In a worldwide perspective, there are hints in the literature that in ~20% of cases, there is a strong tendency to trust an abnormal NIPT result so much that a second test is skipped and termination of the pregnancy is simply based on a single screening test [18,29]. Such policies are also reinforced by the cost-effectiveness of NIPT analyses, which inappropriately encourage NIPT use as a diagnostic versus a screening tool [30].

As recently summarized, all of these nine points must be considered and should be covered in pre-test counselling for everyone considering a NIPT. Therefore medical specialists working with and offering NIPT need to be aware of these peculiarities [14,18,31]. It is surprising that most NIPT related publications do not discuss any of these points and instead provide an overall astonishingly positive view on NIPT. Examples similar to the two following excerpts can be easily found in dozens of NIPT papers:

*(1) “The high specificity, efficiency and safety (non-invasiveness) of NIPT can effectively improve the detection rate of common chromosomal aneuploidy, thereby reducing the occurrence of birth defects”* [32]*; or (2) “NIPT has revolutionized the approach to prenatal diagnosis and, to date, it is the most superior screening method for the common autosomal aneuploidies”*. [33]

## 5. NIPT in Germany

NIPT was introduced in Germany shortly after the first commercial provider brought it to the market in 2012. The first company offering NIPT in Germany even managed to spread the news advertising this new test in the major evening news program on television (the Tagesschau), extensively praising the test as the major medical breakthrough of the century, following its introduction by the end of 2012. Lobbying continued, and while FTS is still not reimbursed by insurance companies in Germany, from 2022 onwards, NIPT is covered under certain conditions:

“*Although the NIPT is part of the statutory health insurance benefits, it is not one of the generally recommended preventive examinations for all pregnant women. This has been repeatedly emphasized by the Federal Joint Committee, which decides on health insurance benefits. The fund pays for the test if there are indications of a trisomy, such as a conspicuous ultrasound, or if “a woman, together with her doctor, comes to the conclusion that the test is necessary in her personal situation”, according to the information for insured persons. Maternal age alone - often a risk factor for a trisomy—is not yet a reason for a NIPT*”.

(translated from [34])

In an official statement, the German health insurance companies declared:

“*The trisomy test NIPT has been included in the statutory benefits catalog of health insurance companies. Only specialists in gynecology and obstetrics with the qualification “specialized genetic counseling, as well as specialists in human genetics” are allowed to perform the NIPT test after intensive counseling. The NIPT is not part of the generally recommended screening examinations during pregnancy. It is only paid for by health insurance companies if the doctor and patient decide together, after intensive counseling, that the trisomy test makes sense in the personal situation of the pregnant woman. The trisomy test can be useful, especially if there are indications of trisomies through the normal screening examinations or as part of the FTS*”.

(translated from [35])

With regard to the advantages I to V of NIPT, mentioned in chapter 3, all of them hold true for Germany, apart from “advantage II”. Germany not only has an excellent network of centers offering invasive diagnostics, it also requires that all citizens residing in Germany take out a health insurance. Correspondingly, each pregnant woman can access the free preventive medical checkups, as regulated in the “Maternity Guidelines” [36]. Thus, NIPT is not necessary to compensate for a lack of invasive diagnostics supply, as exists in other countries.

Taking into consideration the nine critical points of NIPT from chapter 4 (a to i), each is addressed separately below:

for (a) It is not clear to the German public that cffDNA tested in NIPT derives from the placenta [34]; even the NIPT-specific German Genetic Diagnostics Commission (GEKO)-paper does not mention this fact explicitly [37]. In addition, not all German gynecologists working with NIPT, since its introduction, have this issue and the implications in mind [14].
(b) German women are not always aware that a positive NIPT must be tested invasively [14,25], and
(c) a normal NIPT result, is often inappropriately used synonymously with the statement ‘a healthy child will be born’ in Germany, as in other parts of the world [14,25].
(d) There is no data for Germany on the number of positive NIPTs, however, the use of NIPT has not led to increases in induced abortions within the last 10 years. There was even a statistically significant decline (Chi squared test: *p* < 0.0001) in induced abortions from 13.69% down to 10.62% of all pregnancies between 2012 to 2021. This decline was only observable in terminations before the 12th w.o.g. from 13.34% down to 10.28% (Table 2). The reason for this remains unclear, but could be due to a false “feeling of security” after getting a normal NIPT result. This would also correlate well with the use of NIPT in Germany, which was previously (2019) used in ~25% of pregnancies [38] and is nowadays between 50 and 75% [39].
(e) ‘No-call’ results are also a problem in Germany, but no detailed data is available.
(f) False positive NIPT results experienced in Germany have been described in a newspaper article in 2020 [25], and negative NIPT results have to be double checked by sonography/FTS to exclude a false negative NIPT result and conditions not tested by the applied NIPT.
(g) unexpected findings for NIPTs evaluating only trisomies 13, 18 and 21 were also observed in the lab of the author.
(h) The fact that women with false positive NIPT results feel that not enough recognition to this issue is available to the public, cannot be understated; views that have also been highlighted in the previously mentioned interviews completed by a journalist [25] that covered 10–15 cases with false positive NIPT results. In Germany there also seems to be a lack of information or awareness by gynecologists and obstetricians on the restrictions of NIPT; and many of them seem to be overtaxed in cases of an abnormal NIPT result and how to guide and counsel the concerned woman. Still, as according to the German Gene Diagnostics Act (GenDG) [42] pre- and post-genetic-test counselling must be offered, through time, more training and adaptations to the special conditions of the test can be expected in clinics. Another interesting point implied by the GenDG is that prenatal genetic diagnostics may only be performed for medical purposes, i.e., exclusively targeting disease-relevant genetic characteristics. However, most prenatal diagnostic measures do not have a medical benefit in the narrower sense for the fetus, since the diagnosed diseases or variations (for example, trisomy 21) are not yet accessible to prenatal therapy. For other, e.g., obstetric, considerations concerning the welfare of the future child, ultrasound examinations would usually be perfectly adequate. Most procedures also do not have a preventive benefit. Consequently, special demands are made on education and counseling in the context of prenatal genetic diagnostics. The overriding ethical principle in the context of prenatal diagnostics is the reproductive autonomy of the pregnant woman or couple, and this must be supported by the comprehensive information and experience-oriented counseling [43].
(i) According to Table 2, NIPT did not lead to an increase, but instead a decrease of first trimester induced abortions in Germany. An increase was also not expected, as all genetic studies in patients are regulated by GenDG, which among other demands stipulates, that before and after each genetic testing, genetic counselling must be offered. Furthermore, German MDs closely attend, supervise, and examine each pregnant woman regularly; thus, it is not conceivable that an induced abortion could be carried out solely based on an abnormal NIPT. At a minimum, an abnormal sonogram must be present, and in cases where a genetic condition is the rationale for an induced abortion, a council consisting of MDs from different departments, including human genetics must always approve the desire for pregnancy termination (at least after the 12th w.o.g.).

**Table 2:** All pregnancies in Germany, compared to the number/percentages of induced abortions (TOP), according to [40,41].

Year	All Pregnancies	TOP Overall [%]	TOP >12 w.o.g. %	TOP 12–21 w.o.g. %	TOP >21 w.o.g. %
2012	780,359	106,815 [13.69]	13.34	0.30	0.06
2013	784,871	102,802 [13.10]	12.74	0.29	0.07
2014	814,642	99,715 [12.24]	11.90	0.27	0.07
2015	836,812	99,237 [11.86]	11.52	0.26	0.08
2016	890,862	98,721 [11.08]	10.76	0.25	0.07
2017	886,110	101,209 [11.42]	11.11	0.23	0.07
2018	889,509	101,986 [11.47]	11.04	0.24	0.07
2019	878,983	100,893 [11.48]	11.15	0.26	0.07
2020	873,092	99,948 [11.15]	11.11	0.26	0.07
2021	890,088	94,596 [10.62]	10.28	0.27	0.08

Thus, eight of the nine critical points for NIPT from chapter 4 are also valid in Germany, apart from point (i), as discussed. In addition, the following peculiarities must be considered when NIPT is carried out by German MDs.

- The GenDG has been interpreted by the GEKO. Surprisingly, the GEKO classified NIPT not as a “prenatal risk screening” but in opposition to all of the corresponding literature data (see above), as a “prenatal genetic analysis for determining the genetic properties” [36]. This is surprising and the rationale for this conclusion may be attributed to political reasons connected to the introduction of NIPT to the scope of services to be billed via the health insurance funds in 2022.
- According to GenDG, it is valid that “if the sex of an embryo or fetus is determined during a prenatal examination, the pregnant woman may be informed of this after the 12th w.o.g. with her consent” (translated from [44]). Thus, NIPT before the 12th w.o.g. can *per se* only be financed by the insurance companies in Germany if trisomies 13, 18 or 21 are tested exclusively. Other chromosomal imbalances, such as microdeletion and microduplication syndromes are not recommended for evaluation by NIPT in Germany, as they largely result in more invasive procedures in normal fetuses [44].
- Hessel and Henn [45] (2022) also pointed out the following problem: presently it is not possible to select individual NIPT analyses for trisomy 13, 18 or 21, separately. “*Therefore, if a pregnant woman wishes to continue a pregnancy for a child with Down syndrome, because she considers this finding reasonable, she may not realize this*” (translated from [45]). Furthermore, this conundrum interferes with the “*right not to know with regard to parts of the examination result*” defined in the GenDG [42,43]. Given that in the context of all current NIPT services, there is no isolated screening for trisomy 21, the prenatal search for Down syndrome has become a medical standard or at worst even a social norm [45].

Overall, providers of NIPT, which in Germany are exclusively physicians, are well advised to include in their pre-NIPT counselling all 4–5 positive and 11–12 mentioned possibly problematic issues associated with the use of this test. This is most likely impossible to accomplish by gynecologists and obstetricians, and owing to the small number of specialists in human genetics, these extensive counselling needs cannot at present be covered in Germany. A way to address this issue may be the long demanded accreditation of clinical laboratory geneticists [46] into genetic counselling [47]. Interestingly, some German politicians are critical of the test as well, and recently formed a “group of delegates on prenatal diagnostics” (Abgeordnetengruppe zur Pränataldiagnostik) [48]. They stated that

“*We are only at the beginning of a worrisome development because more tests for genetic dispositions are in development and are about to be approved*”; and “*We are united in our conviction that prenatal screening for trisomy 21, 18 and 13 and others must not become routine in pregnancy under any circumstances*”.

(translated from [48])

## 6. Conclusions

The theoretical possibilities of NIPT are promising. However, the problems and limitations must also be considered. Overall, NIPT results are not well understood and the reliability of the obtained data is, at the least, also not communicated well to the MDs ordering the test nor to the pregnant women taking the test. As stated by Hessel and Henn, recently: “*Fact is, that the NIPT is perceived by many pregnant as a standard measure for pregnant women and is carried out unquestioningly*” (translated from [45]). This attitude can also be seen in Germany and may lead to a rude awakening [25]. Thus, appropriate training and continuing education of the physicians providing NIPT counseling [14] are urgently necessary. As outlined elsewhere [14] this can only be achieved by unbiased offers of ongoing education—not from NIPT providers, but from medical associations or independent researchers. Finally, it must be stated that the number of invasive diagnostics decreased in Germany for AC from 20,639 in 2012 to 8538 in 2018 (by 41%) [49]. If this trend continues, many providers of cytogenetic analyses will have to stop their service, according to the legal regulations [50].

## Figures and Tables

**Table 1:** Overview on the presently available invasive and non-invasive prenatal diagnostic tests.

Type of Test	Invasive	
	Can be Carried out from w.o.g.:	Is the Fetus Studied?
(cyto)genetics from CVS	~11	No
(cyto)genetics from AC	~14	Yes
(cyto)genetics from UCB	~20	Yes
**Type of test**	**Non-invasive**	
	**Can be carried out from w.o.g.:**	**Is the fetus studied?**
Early sonography	~6	(Yes) ^1^
Late fine sonography	~19	(Yes) ^1^
First trimester-screening (FTS)	11–14	(No) ^2^
Tests for protein markers (e.g., alpha fetoprotein, etc.) from maternal blood serum	~11	No
Non-invasive prenatal testing (NIPT) from free placenta-derived DNA in maternal blood serum	~10	No
Molecular genetic/molecular cytogenetic tests on fetal nucleated erythrocytes from maternal blood	before 10	Yes

^1^ In sonographic studies, the fetus is observed visually, but no genetic material is studied—thus here ‘Yes’ is given in brackets. ^2^ FTS includes sonography—thus here ‘No’ is given in brackets.

## Data Availability

All of the data this paper is based on is provided or cited in this article itself.

## Supplementary Materials

The following supporting information can be downloaded at: https://www.mdpi.com/article/10.3390/diagnostics12112816/s1, Figure S1: Screenshot of the search in https://www.google.de/ for “invasive prenatal diagnostic risks” giving 2–4 percent for CVS via the cervix and 1–2 percent for CVS via the abdominal wall (search carried out on 16 September 2022).
