# Supplementary figures and images for: Non-Invasive Prenatal Testing in Germany
Source: Diagnostics (Basel). 2022 Nov 16;12(11):2816. doi: 10.3390/diagnostics12112816 (PMC9689121; doi:10.3390/diagnostics12112816)

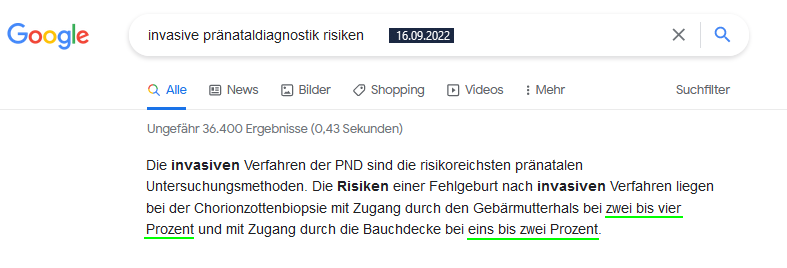

Supplement: Supplementary file 1 [file diagnostics-12-02816-s001.zip › diagnostics-2024196-supplementary.tif]
